# Empagliflozin, Health Status, and Quality of Life in Patients With Heart Failure and Preserved Ejection Fraction: The EMPEROR-Preserved Trial

**DOI:** 10.1161/CIRCULATIONAHA.121.057812

**Published:** 2021-11-15

**Authors:** Javed Butler, Gerasimos Filippatos, Tariq Jamal Siddiqi, Martina Brueckmann, Michael Böhm, Vijay K. Chopra, João Pedro Ferreira, James L. Januzzi, Sanjay Kaul, Ileana L. Piña, Piotr Ponikowski, Sanjiv J. Shah, Michele Senni, Ola Vedin, Subodh Verma, Barbara Peil, Stuart J. Pocock, Faiez Zannad, Milton Packer, Stefan D. Anker

**Affiliations:** Department of Medicine, University of Mississippi School of Medicine, Jackson (J.B., T.J.S.).; National and Kapodistrian University of Athens School of Medicine, Athens University Hospital Attikon, Greece (G.F.).; Boehringer Ingelheim International GmbH, Ingelheim, Germany (M. Brueckmann).; Faculty of Medicine Mannheim, University of Heidelberg, Mannheim, Germany (M. Brueckmann).; Klinik für Innere Medizin III, Universitätsklinikum des Saarlandes, Saarland University, Homburg/Saar, Germany (M. Böhm).; Max Superspeciality Hospital, Saket, New Delhi, India (V.K.C.).; Université de Lorraine, Inserm, Centre d’Investigations Cliniques, Plurithématique 14-33, and Inserm U1116, CHRU, F-CRIN INI-CRCT (Cardiovascular and Renal Clinical Trialists), Nancy, France (J.P.F., F.Z.).; Cardiovascular Research and Development Center, Department of Surgery and Physiology, Faculty of Medicine of the University of Porto, Portugal (J.P.F.).; Massachusetts General Hospital and Baim Institute for Clinical Research, Boston (J.L.J.).; Cedars-Sinai Medical Center, Los Angeles, CA (S.K.).; Central Michigan University, Mount Pleasant (I.L.P.); Wroclaw Medical University, Poland (P.P.).; Northwestern University Feinberg School of Medicine, Chicago, IL (S.J.S.).; Cardiovascular Department, Cardiology Division, Papa Giovanni XXIII Hospital, Bergamo, Italy (M.S.).; Boehringer Ingelheim AB, Stockholm, Sweden (O.V.).; Division of Cardiac Surgery, St. Michael’s Hospital, University of Toronto, ON, Canada (S.V.).; Boehringer Ingelheim Pharma GmbH & Co KG, Ingelheim, Germany (B.P.).; Department of Medical Statistics, London School of Hygiene and Tropical Medicine, United Kingdom (S.J.P.).; Baylor Heart and Vascular Institute, Baylor University Medical Center, Dallas, TX (M.P.).; Imperial College, London, United Kingdom (M.P.).; Department of Cardiology and Berlin Institute of Health Center for Regenerative Therapies, Germany (S.D.A.).; German Centre for Cardiovascular Research partner site Berlin, Germany (S.D.A.).; Charité Universitätsmedizin Berlin, Germany (S.D.A.).

**Keywords:** empagliflozin, health status, heart failure, diastolic, quality of life

## Abstract

**Background:**

Patients with heart failure with preserved ejection fraction have significant impairment in health-related quality of life. In the EMPEROR-Preserved trial (Empagliflozin Outcome Trial in Patients With Chronic Heart Failure With Preserved Ejection Fraction), we evaluated the efficacy of empagliflozin on health-related quality of life in patients with heart failure with preserved ejection fraction and whether the clinical benefit observed with empagliflozin varies according to baseline health status.

**Methods:**

Health-related quality of life was measured with the Kansas City Cardiomyopathy Questionnaire (KCCQ) at baseline and 12, 32, and 52 weeks. Patients were divided by baseline KCCQ Clinical Summary Score (CSS) tertiles, and the effect of empagliflozin on outcomes was examined. The effect of empagliflozin on KCCQ-CSS, Total Symptom Score, and Overall Summary Score was evaluated. Responder analyses were performed to compare the odds of improvement and deterioration in KCCQ related to treatment with empagliflozin.

**Results:**

The effect of empagliflozin on reducing the risk of time to cardiovascular death or heart failure hospitalization was consistent across baseline KCCQ-CSS tertiles (hazard ratio, 0.83 [95% CI, 0.69–1.00], 0.70 [95% CI, 0.55–0.88], and 0.82 [95% CI, 0.62–1.08] for scores <62.5, 62.5–83.3, and ≥83.3, respectively; *P* trend=0.77). Similar results were seen for total heart failure hospitalizations. Patients treated with empagliflozin had significant improvement in KCCQ-CSS versus placebo (+1.03, +1.24, and +1.50 at 12, 32, and 52 weeks, respectively; *P*<0.01); similar results were seen for Total Symptom Score and Overall Summary Score. At 12 weeks, patients on empagliflozin had higher odds of improvement ≥5 points (odds ratio, 1.23 [95% CI, 1.10–1.37]), ≥10 points (odds ratio, 1.15 [95% CI, 1.03–1.27]), and ≥15 points (odds ratio, 1.13 [95% CI, 1.02–1.26]) and lower odds of deterioration ≥5 points in KCCQ-CSS (odds ratio, 0.85 [95% CI, 0.75–0.97]). A similar pattern was seen at 32 and 52 weeks, and results were consistent for Total Symptom Score and Overall Summary Score.

**Conclusions:**

In patients with heart failure with preserved ejection fraction, empagliflozin reduced the risk for major heart failure outcomes across the range of baseline KCCQ scores. Empagliflozin improved health-related quality of life, an effect that appeared early and was sustained for at least 1 year.

**Registration:**

URL: https://www.clinicaltrials.gov; Unique identifier: NCT03057951.

## Abstract 2

Supplemental Digital Content is available in the text.

**Clinical Perspective**

**What Is New?**

- In the EMPEROR-Preserved trial (Empagliflozin Outcome Trial in Patients with Chronic Heart Failure With Preserved Ejection Fraction), baseline health status and quality of life did not influence the magnitude of the effect of empagliflozin on the risk of cardiovascular death or hospitalization for heart failure.
- Empagliflozin improved health status and quality of life, as assessed by the Kansas City Cardiomyopathy Questionnaire, across all domains and at all measured time points (12, 32, and 52 weeks).

**What Are the Clinical Implications?**

- These findings indicate that the ability of sodium glucose cotransporter-2 inhibition with empagliflozin to improve health status and quality of life in patients with a reduced ejection fraction (previously demonstrated in the EMPEROR-Reduced trial [Empagliflozin Outcome Trial in Patients with Chronic Heart Failure With Reduced Ejection Fraction]) extends to patients with a preserved ejection fraction.

Approximately half of all patients with heart failure (HF) have preserved ejection fraction (HFpEF).^1,2^ Not only do patients with HFpEF experience similar risk for adverse clinical outcomes compared with those with HF with reduced ejection fraction, but also both HF phenotypes have similarly impaired physical functioning and health-related quality of life (HRQoL).^3,4^ Although the overall burden of impaired HRQoL is similar in both HF with reduced ejection fraction and HFpEF, most of the data related to health status in HF have been derived from patients with HF with reduced ejection fraction.^5,6^

The EMPEROR-Preserved trial (Empagliflozin Outcome Trial in Patients with Chronic Heart Failure with Preserved Ejection Fraction) studied the sodium glucose cotransporter-2 inhibitor empagliflozin in patients with HFpEF and a left ventricular ejection fraction >40% and showed a significant reduction in the risk of cardiovascular death or HF hospitalization.^7^ The overall patient’s health status, including HRQoL, in the EMPEROR-Preserved trial was assessed with the Kansas City Cardiomyopathy Questionnaire (KCCQ), providing an opportunity to understand the impact of baseline HRQoL on clinical benefits with empagliflozin and, conversely, the effect of empagliflozin on HRQoL.

## Methods

### Study Design and Patient Population

The design and primary results of the EMPEROR-Preserved trial have been published previously.^6^ In brief, the EMPEROR-Preserved trial was a phase III international, multicenter, randomized, double-blind, parallel-group, placebo-controlled trial that enrolled adult patients who had chronic HF with New York Heart Association class II to IV symptoms for at least 3 months and a left ventricular ejection fraction of >40% with no previous measurement of ≤40% under stable conditions. Patients were required to have elevated NT-proBNP (N-terminal pro-B-type natriuretic peptide) levels (>900 pg/mL or >300 pg/mL in patients with or without atrial fibrillation, respectively) and evidence of structural heart disease (left ventricular hypertrophy or left atrial enlargement) or a documented hospitalization for HF within the 12 months before enrollment. The protocol was approved by the ethics committee of each of the 622 participating sites in 23 countries, and all patients gave written informed consent.

### Quality of Life Outcome Assessment

HRQoL was assessed with KCCQ-23, which includes 23 items that map to 7 domains: symptom frequency; symptom burden and stability; physical limitations; social limitations; quality of life; and self-efficacy. The KCCQ scores are summarized as (1) Total Symptom Score (TSS), which consists of symptom frequency and symptom burden domains; (2) Clinical Summary Score (CSS), consisting of physical limitation and TSS; and (3) Overall Summary Score (OSS), which is formed by combining CSS, quality of life, and social limitation domains. The scores range from 0 to 100, with 100 being the best possible score. The KCCQ has been shown to be valid, reliable, and sensitive to clinical changes, and lower KCCQ scores are associated with higher risk of hospitalizations and mortality.^8–10^ The KCCQ was completed by patients at baseline and at 12, 32, and 52 weeks after randomization to placebo or empagliflozin.

### Statistical Analysis

Study participants were categorized according to tertiles of baseline KCCQ-CSS, KCCQ-TSS, and KCCQ-OSS. Baseline characteristics were summarized as frequencies and percentages or means with SDs. The effect of empagliflozin in each tertile was assessed by hazard ratios with 95% CIs using a Cox proportional hazard model, accounting for noncardiovascular death as a competing risk. In addition, the effect of empagliflozin on total (first and recurrent) hospitalizations for HF in KCCQ tertiles was analyzed by a joint frailty model with cardiovascular death as a competing risk.

To assess the affect of empagliflozin on HRQoL, differences between treatment groups in mean KCCQ-CSS, KCCQ-TSS, and KCCQ-OSS at 12, 32, and 52 weeks were calculated with a mixed model for repeated measures, and the least-squares mean difference between treatment groups was estimated after adjustment for baseline KCCQ score, estimated glomerular filtration rate, age, region, sex, diabetes status, and left ventricular ejection fraction. Responder analyses were performed to investigate the proportion of patients with an improvement or deterioration in KCCQ score at 12, 32, and 52 weeks after randomization; established clinically meaningful thresholds for changes in KCCQ (≥5, ≥10, and ≥15 points for improvement and ≥5 point for deterioration) were used for all responder analyses.

Multiple imputation was used to account for missing KCCQ values, and estimates were combined by use of the Rubin rules.^11^ Odds ratios with 95% CIs were calculated from a logistic regression model, which included baseline KCCQ score, estimated glomerular filtration rate, age, region, sex, diabetes status, and ejection fraction as covariates. Patients who died before 12, 32, and 52 weeks were counted as not improved in the analyses of improvement and as worse in the analyses of deterioration. To accommodate for the fact that patients with a very high baseline KCCQ score are not able to experience certain numeric improvements, patients with baseline KCCQ values of ≥95, ≥90, or ≥85 points in KCCQ domains were considered to have 5-, 10-, or 15-point improvement, respectively, if their values remained ≥95, 90, or 85. Similarly, patients with a KCCQ score ≤5 points at baseline were defined as deteriorated if their score remained ≤5 points. All analyses were conducted with SAS version 9.4 (SAS Institute, Cary, NC).

### Data Sharing

The sponsor of the EMPEROR-Preserved trial (Boehringer Ingelheim) is committed to responsible sharing of clinical study reports, related clinical documents, and patient-level clinical study data. Researchers are invited to submit inquiries via the Boehringer Ingelheim website.

## Results

### Patient Population

Among the 5942 participants with a baseline KCCQ assessment, the mean (SD) KCCQ-CSS, KCCQ-TSS, and KCCQ-OSS were 70.4 (21.2), 73.5 (22.0), and 68.9 (21.1), respectively. Baseline characteristics of patients in KCCQ-CSS tertiles are shown in Table 1. Patients with lower KCCQ-CSS were more often female and White, were more often enrolled in Europe, and were more likely to have worse New York Heart Association class, higher body mass index and NT-proBNP levels, and a history of diabetes and atrial fibrillation. An overview of the availability of KCCQ-CSS data at each time point is shown in Figure S1.

**Table 1.**
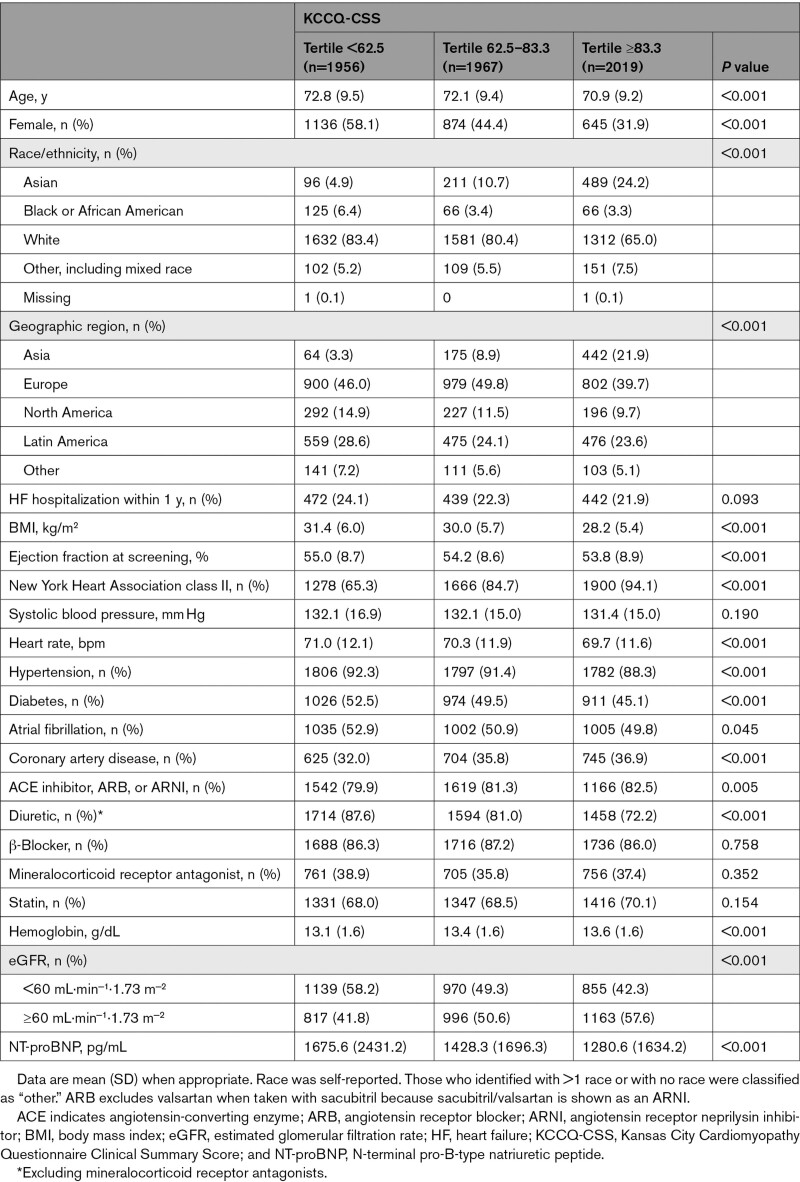
Baseline Characteristics According to KCCQ-CSS at Baseline

### Effect of Baseline HRQoL on Benefit With Empagliflozin

**Figure 1.**
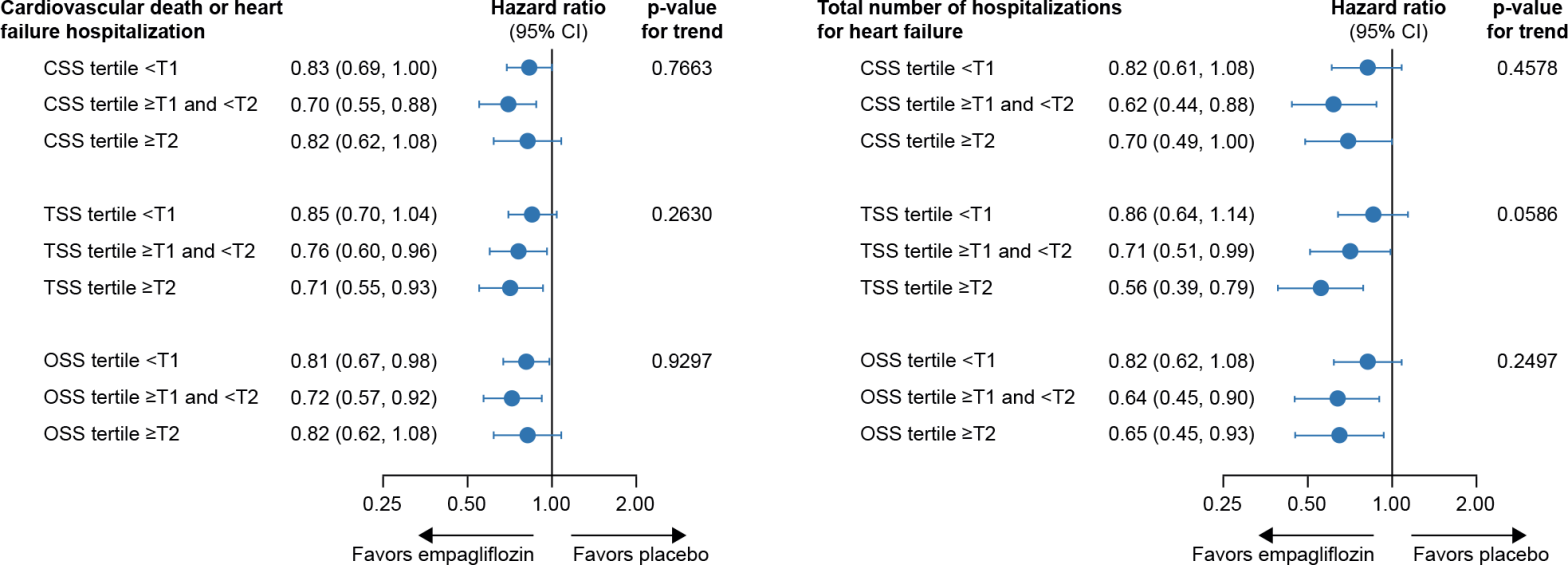
**Effect of empagliflozin on outcomes by baseline KCCQ tertiles.** CSS indicates Clinical Summary Score; KCCQ, Kansas City Cardiomyopathy Questionnaire; OSS, Overall Summary Score; and TSS, total symptom score. *P value from trend test assuming ordering of the KCCQ tertiles

Empagliflozin reduced the primary outcome of time to cardiovascular death or HF hospitalization across the entire range of KCCQ-CSS (hazard ratio, 0.83 [95% CI, 0.69–1.00], 0.70 [95% CI, 0.55–0.88], and 0.82 [95% CI, 0.62–1.08] for patients with baseline scores <62.5, 62.5–83.3, and ≥83.3, respectively; *P* trend=0.77; Figure 1 and Figure S2). Similar results were observed for KCCQ-TSS and KCCQ-OSS. Empagliflozin reduced the total number of HF hospitalizations in each of the KCCQ-CSS tertiles (hazard ratio, 0.82 [95% CI, 0.61–1.08], 0.62 [95% CI, 0.44–0.88], and 0.70 [95% CI, 0.49–1.00] for scores <62.5, 62.5–83.3, and ≥83.3, respectively; *P* trend=0.46). Results were similar for KCCQ-OSS and KCCQ-TSS (Figure 1).

### Effect of Empagliflozin on HRQoL

**Figure 2.**
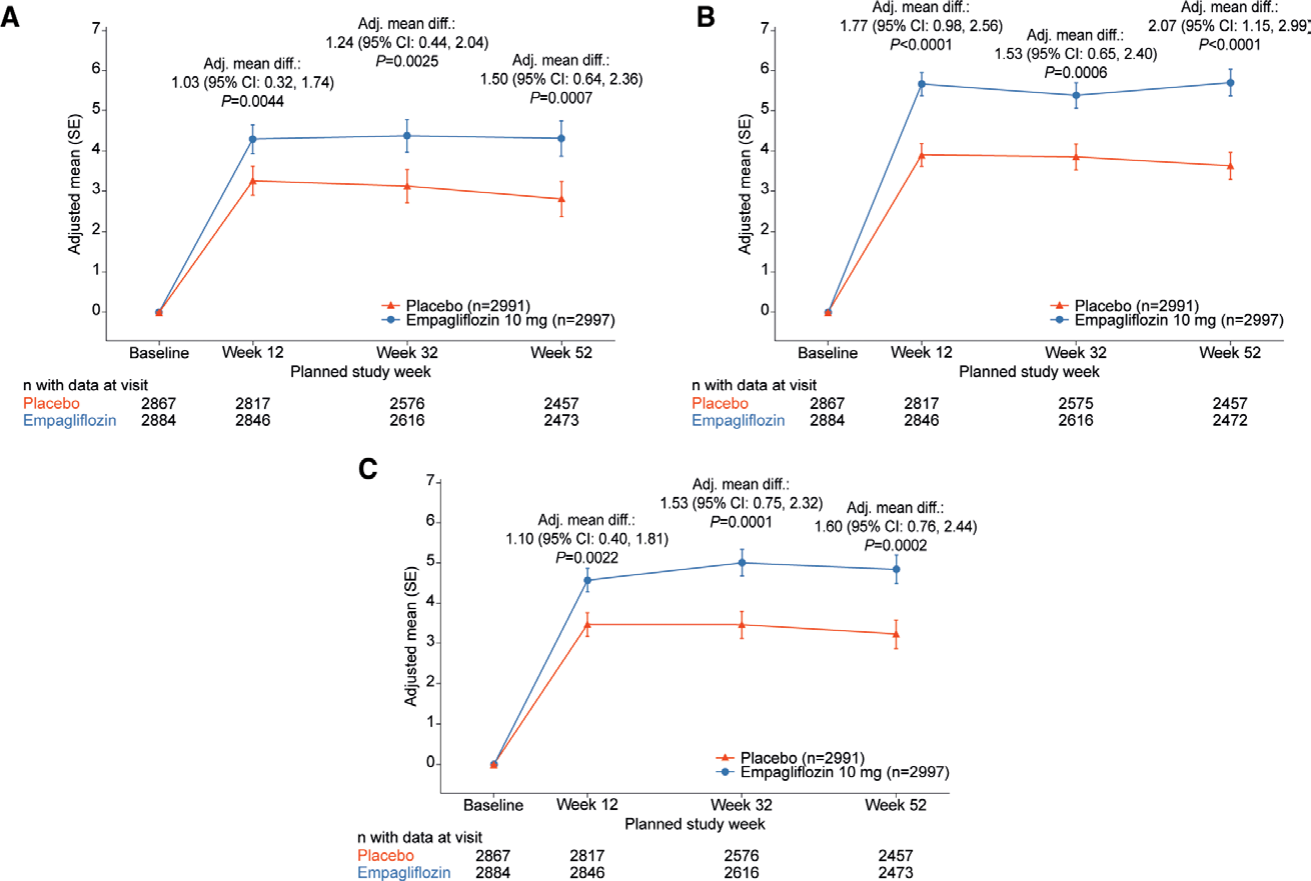
**Effects of empagliflozin versus placebo on mean KCCQ scores.** Changes in (**A**) Kansas City Cardiomyopathy Questionnaire (KCCQ) Clinical Summary Score, (**B**) Total Symptom Score, and (**C**) Overall Summary Score from baseline to 12, 32, and 52 weeks for empagliflozin versus placebo. Adj. mean diff indicates adjusted mean difference.

**Figure 3.**
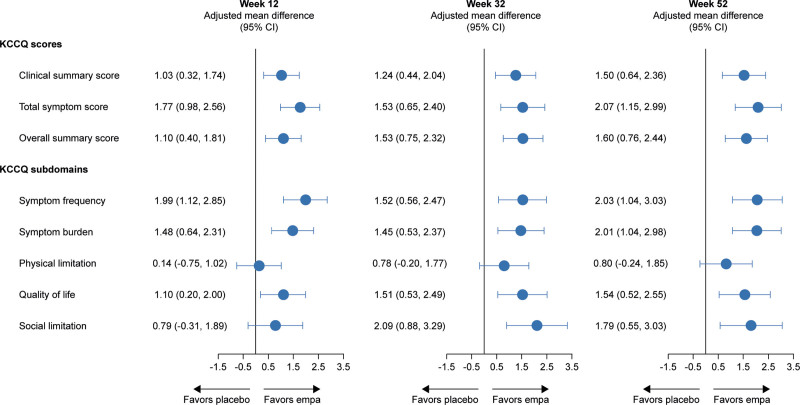
**Adjusted mean difference in KCCQ-CSS, TSS, OSS, and subdomains for empagliflozin versus placebo at 12, 32, and 52 weeks.** CSS indicates Clinical Summary Score; empa, empagliflozin; KCCQ, Kansas City Cardiomyopathy Questionnaire; OSS, Overall Summary Score; and TSS, Total Symptom Score.

The adjusted mean change in KCCQ-CSS, KCCQ-TSS, and KCCQ-OSS by treatment arms over time is presented in Figure 2A through 2C. Compared with those treated with placebo, patients treated with empagliflozin had a significant improvement in mean KCCQ score at 12, 32, and 52 weeks: CSS, 1.03, 1.24, and 1.50 points; TSS, 1.77, 1.53, and 2.07 points; and OSS, 1.10, 1.53, and 1.60 points, respectively (*P*<0.01 for all; Figure 3). The effect of empagliflozin on KCCQ-CSS, KCCQ-TSS, and KCCQ-OSS by tertiles of baseline score at 12, 32, and 52 weeks is shown in Table 2.

**Table 2.**
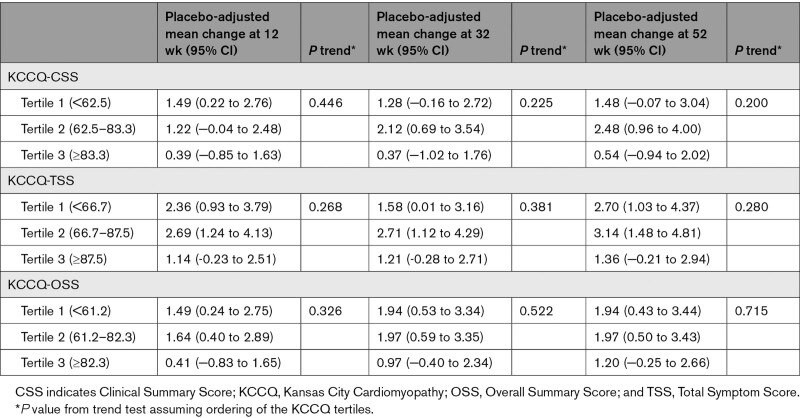
Effect of Empagliflozin on KCCQ Scores at 12, 32, and 52 Weeks

**Figure 4.**
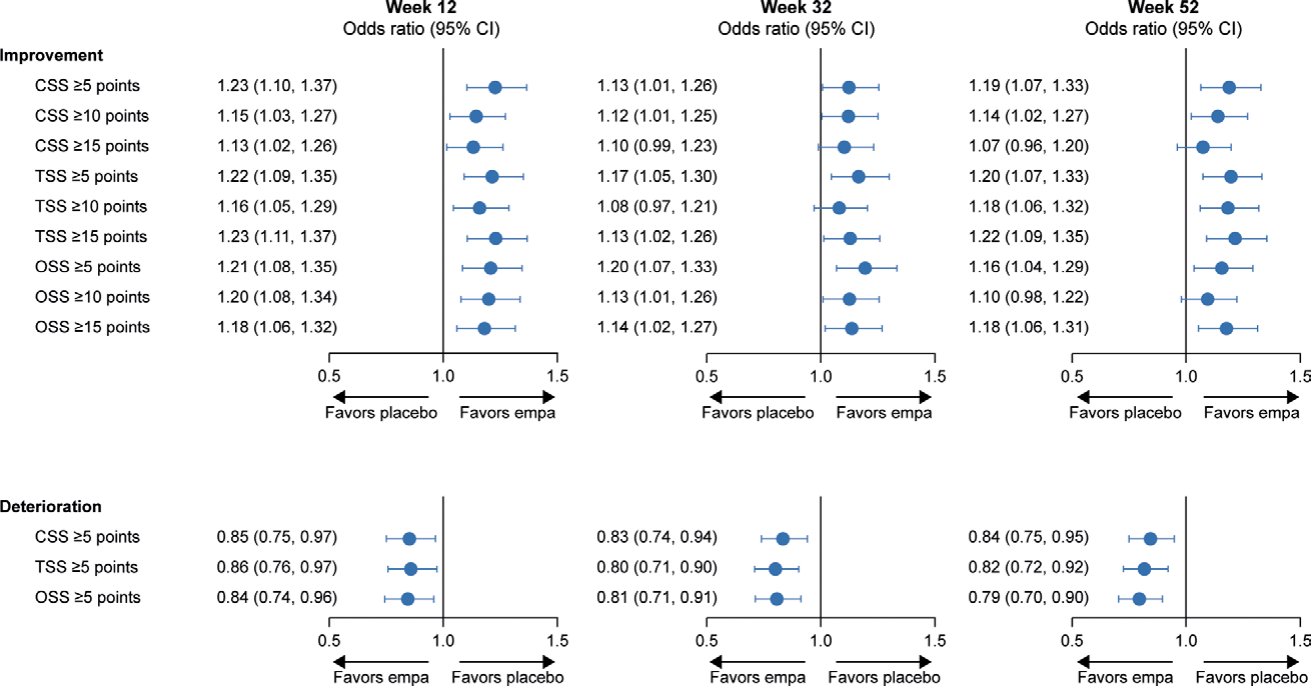
**Responder analysis for improvement and deterioration across the KCCQ domains.** CSS indicates Clinical Summary Score; empa, empagliflozin; KCCQ, Kansas City Cardiomyopathy Questionnaire; OSS, Overall Summary Score; and TSS, total symptom score

**Figure 5.**
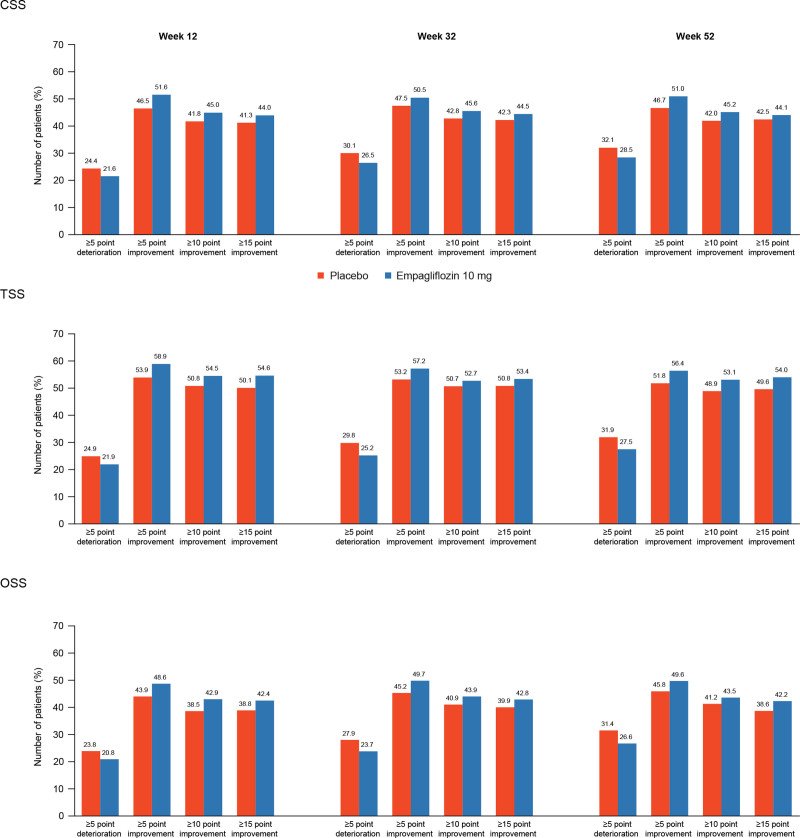
**Responder analysis with proportion of responders at 12, 32, and 52 weeks with empagliflozin versus placebo.** CSS indicates Clinical Summary Score; OSS, Overall Summary Score; and TSS, Total Symptom Score.

At 12 weeks, patients in the empagliflozin arm were more likely to show meaningful improvements (≥5 points [51.6% versus 46.5%], ≥10 points [45.0% versus 41.8%], ≥15 points [44.0% versus 41.3%]) and less likely to show deterioration (≥5 points [21.6% versus 24.4%]) in KCCQ-CSS. The odds ratios for the effect of empagliflozin versus placebo at 12 weeks were 1.23 (95% CI, 1.10–1.37) with a number needed to treat of 20 (95% CI, 14–40) for a ≥5-point improvement, 1.15 (95% CI, 1.03–1.27) with a number needed to treat of 31 (95% CI, 18–140) for a ≥10-point improvement, and 1.13 (95% CI, 1.02–1.26) with a number needed to treat of 38 (95% CI, 20–708) for a ≥15-point improvement. The odds ratio for the effect of empagliflozin for a ≥5-point deterioration was 0.85 (95% CI, 0.75–0.97) with a number needed to treat of 35 (95% CI, 20–138). Similar trends were observed at 32 and 52 weeks, and results were generally consistent for KCCQ-TSS and KCCQ-OSS (Figures 4 and 5).

## Discussion

In this prespecified analysis of the EMPEROR-Preserved trial, we show 2 key findings. First, empagliflozin reduced the risk for major HF outcomes in patients with HFpEF across the entire range of baseline HRQoL. Second, empagliflozin improved HRQoL, and the improvement was seen early and was sustained for at least 1 year. Patients treated with empagliflozin were more likely to show clinically meaningful improvement and less likely to experience clinically meaningful deterioration in health status compared with patients treated with placebo. These findings are highly concordant with those reported with empagliflozin in patients with a reduced ejection fraction (≤40%) who were enrolled in the EMPEROR-Reduced trial (Empagliflozin Outcome Trial in Patients With Chronic Heart Failure With Reduced Ejection Fraction).^5^ Together, these data suggest that empagliflozin improves HRQoL across a broad range of patients with HF.

Several studies have assessed the effect of treatment on health status in patients with HFpEF.^12–17^ The TOPCAT trial (Treatment of Preserved Cardiac Function Heart Failure With an Aldosterone Antagonist) with 3400 patients showed a baseline mean KCCQ-OSS of 54.8 and demonstrated a 1.36-point improvement over placebo at 4 months.^12^ The PARAGON-HF trial (Prospective Comparison of ARNI With ARB Global Outcomes in HFpEF) enrolled patients with a baseline health status similar to that in EMPEROR-Preserved (mean KCCQ-CSS, 74.2) and showed an improvement in KCCQ-CSS with sacubitril/valsartan by 1.0 point compared with placebo at 8 months.^13^ Several smaller trials have also assessed the effect of treatments on KCCQ in patients with HFpEF. The VITALITY-HFpEF trial (Patient-Reported Outcomes in Vericiguat-Treated Patients With HFpEF) showed no improvement in KCCQ with vericiguat.^14^ In the NEAT-HFpEF trial (Nitrate’s Effect on Activity Tolerance in Heart Failure With Preserved Ejection Fraction), treatment with isosorbide mononitrate showed numerically (although not statistically significant) unfavorable changes in KCCQ scores.^15^ The EMPERIAL-Preserved trial (Empagliflozin Outcome Trial in Patients With Chronic Heart Failure With Preserved Ejection Fraction) did not show a significant effect of empagliflozin on KCCQ-TSS in a 12-week trial in ≈300 mildly symptomatic patients with HFpEF.^17^ In contrast, the PRESERVED-HF trial (Dapagliflozin in Preserved Ejection Fraction Heart Failure) showed a significant improvement in the KCCQ-CSS with dapagliflozin in patients with HFpEF^16^; the trial enrolled patients with obesity in the United States with >40% having New York Heart Association class III to IV symptoms.

The magnitude of the treatment effect on KCCQ health status seen in the EMPEROR-Preserved trial may appear to be modest (1.0–2.0 points) compared with a change of 5.0 points, which is commonly regarded as representing a clinically meaningful shift in KCCQ scores. However, the 5-point threshold change has been identified as meaningful in individual patients rather than in populations of patients.^18^ In population studies, it may be difficult to achieve a 5-point between-group difference, especially if the baseline KCCQ score is >70, indicative of a reasonably good quality of life and health status. Large between-group differences in KCCQ scores (eg, 10- to 15-point treatment effects) have typically been observed only in patients who were severely compromised at baseline and particularly in unblinded device trials, in which knowledge that a patient has received active therapy likely exaggerated changes in their perception of their own response to an experimental intervention.^19^ Decisions about the handling of missing data and imputation methods may also amplify the size of a treatment difference. It is therefore noteworthy that the magnitude of the treatment effect in EMPEROR-Preserved is similar to that seen in other large-scale double-blind trials of drug therapies, particularly in patients with HFpEF (eg, TOPCAT and PARAGON-HF).^12,13^ Furthermore, our findings with respect to changes in KCCQ scores are concordant with favorable changes in New York Heart Association functional class that we have previously reported in this trial.^20^

Our analyses and findings should be considered in light of certain strengths and limitations. The current study is the largest trial to evaluate the effect of any treatment on health status and quality of life, and our data were complete through 1 year in nearly 90% of patients. Longer-term data were not collected in this trial, but it is often difficult to interpret data beyond 12 months because of competing risks of deaths and other serious events. Furthermore, we studied stable patients who primarily had functional class II symptoms, and treatment effects may have differed if we had enrolled patients with greater degrees of disability and limitation at the start of the trial. Finally, the current analysis did not evaluate the influence of ejection fraction or sex on the effect of empagliflozin on KCCQ scores because these analyses are being presented fully in other publications. If brief, we previously reported an attenuated response for the effect of empagliflozin on HF hospitalizations in patients with ejection fractions ≥60% to 65%,^20^ and we noted an attenuated effect of empagliflozin on KCCQ scores in patients with the highest ejection fractions. In contrast, sex did not influence the effect of empagliflozin on KCCQ scores in the EMPEROR-Preserved trial, whereas in the PARAGON-HF trial, KCCQ scores in men responded significantly more favorably to sacubitril/valsartan than KCCQ scores in women.^21^

## Conclusions

Treatment with empagliflozin reduced the risk for cardiovascular death or HF hospitalization across the range of baseline HRQoL scores in patients with HFpEF. Empagliflozin also significantly improved HRQoL in patients with HFpEF, and this improvement was seen early and was sustained for at least 1 year.

## Article Information

### Acknowledgments

The authors were fully responsible for all content and editorial decisions, were involved at all stages of development, and have approved the final version. Graphical assistance was supported financially by 7.4 Limited.

### Sources of Funding

The EMPEROR-Preserved trial (Empagliflozin Outcome Trial in Patients With Chronic Heart Failure With Preserved Ejection Fraction) was supported by Boehringer Ingelheim and Eli Lilly and Company.

### Disclosures

Dr Butler reports consulting fees from Boehringer Ingelheim, Cardior, CVRx, Foundry, G3 Pharma, Imbria, Impulse Dynamics, Innolife, Janssen, LivaNova, Luitpold, Medtronic, Merck, Novartis, NovoNordisk, Relypsa, Roche, Sanofi, Sequana Medical, V-Wave Ltd, and Vifor. Dr Filippatos reports lectures and/or committee member contributions in trials sponsored by Medtronic, Vifor, Servier, Novartis, Bayer, Amgen, and Boehringer Ingelheim. T.J. Siddiqi has no conflicts of interest to declare. Drs Brueckmann, Vedin, and Peil are employees of Boehringer Ingelheim. M. Böhm is supported by the Deutsche Forschungsgemeinschaft (German Research Foundation; TTR 219, project No. 322900939) and reports personal fees from Abbott, Amgen, AstraZeneca, Bayer, Boehringer Ingelheim, Cytokinetics, Medtronic, Novartis, Servier, and Vifor during the conduct of the study. Dr Chopra reports personal fees from AstraZeneca, Boehringer Ingelheim, and Novartis. Dr Ferreira reports consulting fees from Boehringer Ingelheim during the conduct of the study. Dr Januzzi reports grant support, consulting income, and participation in clinical end point committees/data safety monitoring boards from Janssen; participation in clinical end point committees/data safety monitoring boards from Boehringer Ingelheim; grant support from Novartis, Innolife, Applied Therapeutics, and Siemens Diagnostics; and consultancy fees from Novartis, Roche Diagnostics, and Abbott Diagnostics. Dr Kaul reports personal fees from Boehringer Ingelheim during the conduct of the study and personal fees from AstraZeneca, Janssen Pharmaceuticals, Merck, Novo Nordisk, GSK, Abbott, Amarin, and Novartis outside the submitted work. Dr Piña reports personal fees from Boehringer Ingelheim. Dr Ponikowski reports personal fees from Boehringer Ingelheim, AstraZeneca, Servier, Bristol Myers Squibb, Amgen, Novartis, Merck, Pfizer, and Berlin Chemie, as well as grants and personal fees from Vifor Pharma. Dr Shah has received research grants from the National Institutes of Health (R01 HL107577, R01 HL127028, R01 HL140731, and R01 HL149423), the American Heart Association (No. 16SFRN28780016), Actelion, AstraZeneca, Corvia, Novartis, and Pfizer, as well as consulting fees from Abbott, Actelion, AstraZeneca, Amgen, Axon Therapeutics, Bayer, Boehringer Ingelheim, Bristol Myers Squibb, Cardiora, CVRx, Cytokinetics, Eisai, GSK, Ionis, Ironwood, Lilly, Merck, MyoKardia, Novartis, Novo Nordisk, Pfizer, Regeneron, Sanofi, Shifamed, Tenax, and United Therapeutics. Dr Kaul has received research grants from the American Heart Association (No. 19TPA34890060) and the National Institutes of Health (P30AG059988 and P30DK092939). Dr Senni reports consultancy fees from Abbot, Bayer, Bayer Healthcare, Merck, Novartis, and Vifor Pharma. Dr Verma holds a Tier 1 Canada Research Chair in Cardiovascular Surgery; reports receiving research grants and honoraria from Amarin, Amgen, AstraZeneca, Bayer, Boehringer Ingelheim, Bristol Myers Squibb, Eli Lilly, HLS Therapeutics, Janssen, Novartis, Novo Nordisk, PhaseBio and Pfizer; and reports receiving honoraria from Sanofi, Sun Pharmaceuticals, and the Toronto Knowledge Translation Working Group. He is a member of the scientific excellence committee of the EMPEROR-Reduced trial (Empagliflozin Outcome Trial in Patients with Chronic Heart Failure With Reduced Ejection Fraction) and served as a national lead investigator of the DAPA-HF (Dapagliflozin and Prevention of Adverse Outcomes in Heart Failure) and EMPEROR-Reduced trials. He is the president of the Canadian Medical and Surgical Knowledge Translation Research Group, a federally incorporated not-for-profit physician organization. Dr Pocock reports personal fees from Boehringer Ingelheim, during the conduct of the study. Dr Zannad reports personal fees from Boehringer Ingelheim during the conduct of the study; personal fees from Janssen, Novartis, Boston Scientific, Amgen, CVRx, AstraZeneca, Vifor Fresenius, Cardior, Cereno Pharmaceutical, Applied Therapeutics, Merck, Bayer, and Cellprothera outside of the submitted work; and other support from cardiovascular clinical trialists and Cardiorenal outside of the submitted work. Dr Packer reports personal fees from Boehringer Ingelheim during the conduct of the study and personal fees from Abbvie, Actavis, Amgen, Amarin, AstraZeneca, Boehringer Ingelheim, Bristol Myers Squibb, Casana, CSL Behring, Cytokinetics, Johnson & Johnson, Lilly, Moderna, Novartis, ParatusRx, Pfizer, Relypsa, Salamandra, Synthetic Biologics, and Theravance outside the submitted work. Dr Anker has received grants from Vifor; has received personal fees from Vifor, Bayer, Boehringer Ingelheim, Novartis, Servier, Impulse Dynamics, Cardiac Dimensions, and Thermo Fisher Scientific; and has received grants and personal fees from Abbott Vascular outside the submitted work.

### Supplemental Material

Expanded Methods

Figures S1 and S2

## Supplementary Material


