# Supplementary material for: Empagliflozin, Health Status, and Quality of Life in Patients With Heart Failure and Preserved Ejection Fraction: The EMPEROR-Preserved Trial
Source: Circulation. 2021 Nov 15;145(3):184–93. doi: 10.1161/CIRCULATIONAHA.121.057812 (PMC8763045; doi:10.1161/CIRCULATIONAHA.121.057812)
Supplement: Supplementary file 1 [file cir-145-184-s001.pdf]

SUPPLEMENTAL MATERIALS

Expanded Methods for Multiple Imputation and Correction of Ceiling effect for responder analysis

Handling of missing data: Multiple Imputation

Patients who are alive at 32 weeks and have missing baseline or 32 week KCCQ assessments have their missing CSS imputed using the multiple imputation (MI) methodology as follows. Missing CSS values at baseline or at 32 weeks were imputed under the Missing at Random (MAR) assumption. The imputation was done using a predictive mean matching multiple imputation model and a method of Fully Conditional Specification as implemented in the SAS Procedure MI (FCS statement). The predictive mean matching method ensured that the imputed values remain in the permissible range of the CSS values.

For imputation of baseline CSS the imputation model included the continuous covariates age, baseline LVEF and EGFR at baseline and categorical covariates, sex, diabetes status at baseline and region.

The imputation model for values after baseline included the treatment group, age, baseline EGFR, baseline CSS value, week 12, LVEF at baseline , sex, diabetes status at baseline, region, and a categorical variable representing the number of HHF (0, 1, ≥2 events) in the interval from randomization to 12 week and in the interval from 12 to 32 weeks. Occurrences of HHF are determined based on the investigator reported potential HHF.

100 iterations were used for multiple imputation. For the point estimate of the N and % of patients with an endpoint the mean over all imputations is reported.

Analogue specifications have been used for 12 weeks, 52 weeks and the other summary scores TSS and OSS.

Responder analysis with correction for ceiling effect and handling of death

Number and percentage of patients in each treatment group were summarized across the following categories:

5-point deterioration from baseline to 32 weeks in CSS vs no significant deterioration:

- Death prior to the 32 weeks assessment or change from baseline in CSS ≤ -5 points, vs
- Change from baseline to 32 weeks in CSS > -5 points.

If patients had a baseline value of CSS ≤ 5 points, i.e., near the “floor”, they are defined as having a 5-point deterioration only if they had CSS ≤ 5 points at 32 weeks.

5-point improvement from baseline to 32 weeks in CSS vs no significant improvement:

- Change from baseline in CSS ≥ 5 points, vs
- Death prior to the 32 weeks assessment or change from baseline in CSS < 5 points.

If patients had a baseline value of CSS ≥ 95 points, i.e. near the “ceiling”, they are defined as having a 5 point improvement only if they had CSS ≥ 95 points at 32 weeks.

10-point improvement from baseline to 32 weeks in CSS vs no significant improvement:

- Change from baseline in CSS ≥ 10 points, vs
- Death prior to the 32 weeks assessment or change from baseline in CSS < 10 points.

If patients had a baseline value of CSS ≥ 90 points, they are defined as having a 10 point improvement only if they had CSS ≥ 90 points at 32 weeks.

15-point improvement from baseline to 32 weeks in CSS vs no significant improvement:

- Change from baseline in CSS ≥ 15 points, vs
- Death prior to the 32 weeks assessment or change from baseline in CSS < 15 points.

If patients had a baseline value of CSS ≥ 85 points, they are defined as having a 10 point improvement only if they had CSS ≥ 85 points at 32 weeks.

The proportion of CSS responder categories were compared between treatment groups using a logistic regression model including treatment group, continuous covariates age, LVEF at baseline, EGFR at baseline and baseline CSS value, and categorical covariates sex, diabetes status at baseline and region. Multiple imputations were used to impute missing data. The observed number of and proportion of CSS responders, odds ratio between treatment groups and its 95% confidence interval and corresponding 2-sided p-value estimated from each imputed dataset were combined using Rubin's rule, and the combined results are presented.

Supplemental Figure I: Summary of the availability of Kansas City Cardiomyopathy Questionnaires scores at each time point for the empagliflozin and placebo group.

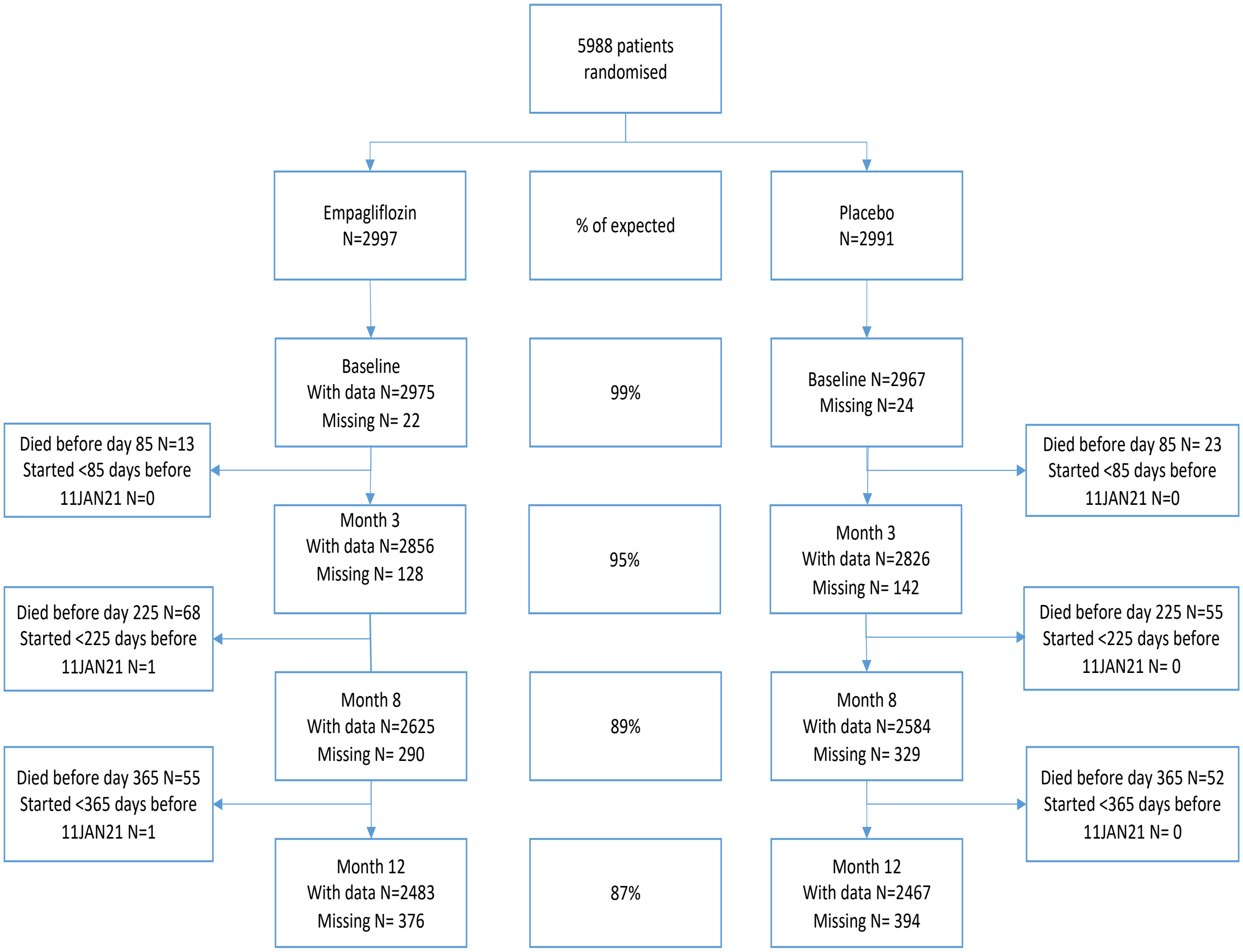

Supplemental Figure II: Cumulative incidence curves depicting **e**ffects of empagliflozin vs. placebo on time to first event of cardiovascular death or heart failure hospitalization according to baseline Kansas City Cardiomyopathy Questionnaires-clinical summary score

Tertile 1

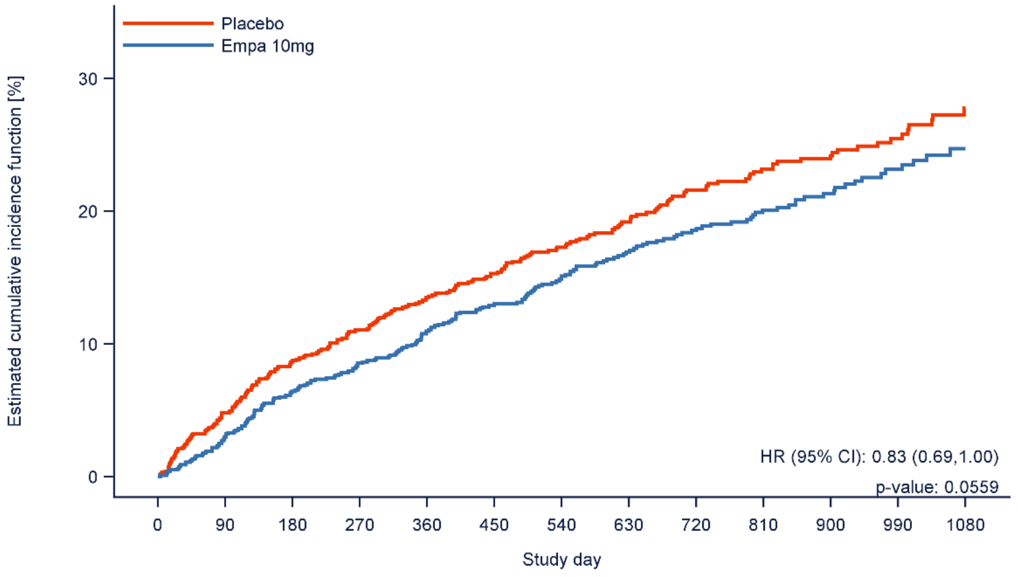

Patients at risk

Placebo	955	903	860	832	806	747	621	544	458	386	302	210	124
Empa 10mg	1001	963	916	892	865	784	660	557	474	402	305	212	116

Tertile 2

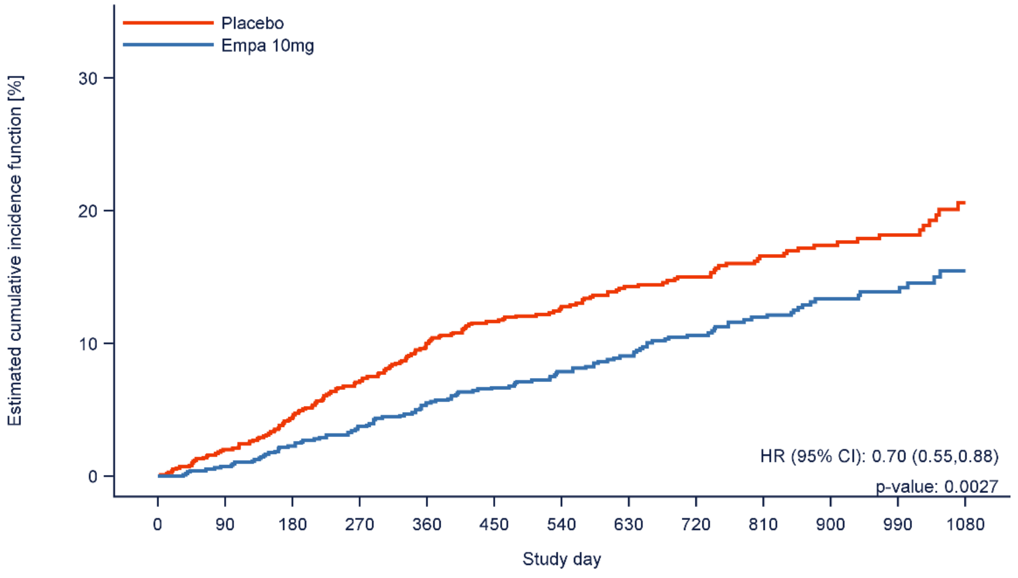

Patients at risk

Placebo	997	968	937	908	874	809	711	616	507	424	322	234	138
Empa 10mg	970	956	933	912	892	821	710	629	540	461	348	242	132

Tertile 3

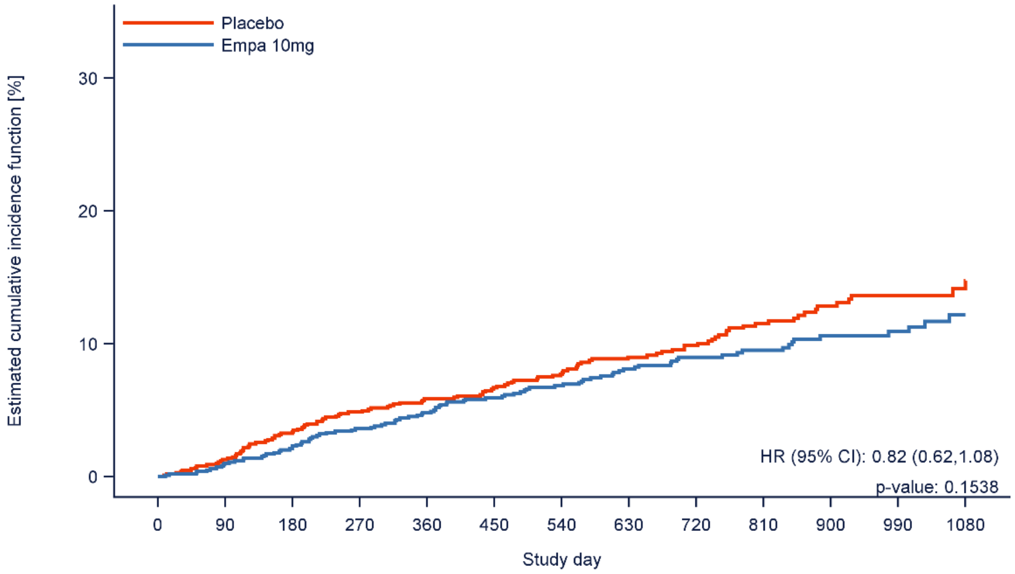

Patients at risk

Placebo	1015	994	971	950	931	855	722	650	558	458	330	231	133
Empa 10mg	1004	988	974	956	931	866	748	658	550	455	341	247	149
